# Field Cookery in the Territorial Force

**Published:** 1911-07-22

**Authors:** 


### The Royal Army Medical Corps Section.

### FIELD COOKERY IN THE TERRITORIAL FORCE.

Apart from its value as a safeguard in counteracting any harmful properties in the food itself, the importance of proper cooking in connection with the health of the Territorial soldier cannot be over-estimated, and as the art of doing it rapidly and well is not difficult of acquisition, all ranks should have a practical knowledge of it.

### The Study of Field Cookery.

It may be pointed out that in the British Army the matter of cooking has of late years very properly received special attention, and there is at Aldershot an Army School of Cookery where there are given throughout the year courses of instruction extending over a period of two to three months. Issued by this Army School of Cookery is a Manual of Military Cooking, and a perusal of it will show how very fully the subject is dealt with in these instructional courses. To every regiment of the Regular Army there is posted a master-cook, with the rank of sergeant, holding a certificate of the Army School of Cookery. He trains about two men per company as assistant cooks, and they remain permanently in this capacity, being freed from all other regimental duties. As long as a regiment is stationed in barracks the cooking for the men is of course much facilitated by there being available different recognised apparatus, such as that of Warren, Dean, and Richmond. With the aid of these it is an easy matter to vary the diet, so that each company may have a complete change daily throughout the week, but the conditions are very different when the regiment has to go into camp or is ordered on active service in the field.

### The Apparatus.

To meet the altered situation what is known as "Field cooking" has to be resorted to, and for this purpose special apparatus is provided. It consists of camp or service kettles of large size, each of which holds twelve quarts and is able to cook for eight men with vegetables and fifteen without. To each regiment seventy-two such kettles and a Soyer's stove are issued, and with these all the cooking in camp has to be done. They are oval in shape and in these boiling, stewing, and frying can be carried out, while they serve admirably for making tea, one kettle being sufficient for twenty-four men at the rate of a pint each. To allow of cooking being carried on with these kettles some form of kitchen must be improvised, and it may take the form of a "broad arrow," or "trench," or "wall kitchen," or an "Aldershot gridiron." The type selected will depend on the length of time the encampment is for. If only for the night, the "trench" kitchen is the best and serves admirably. It is about seven and a half feet long, nine inches wide, and eighteen inches deep, and on its sides seven of the large oval service kettles can be placed. The fuel used is generally wood and it is placed in the trench. It is obtained from the Army Service Corps and is issued at the rate of 3 lb. per man.

### The Question of Fuel.

With the above-mentioned equipment and by these extemporised kitchens cooking can be very well carried on in encampments, but when the troops are on the move and in forced marches the chief and initial difficulty in field cooking is the carriage of fuel sufficient for all arms. In these circumstances recourse must be had to the canteen or mess-tin that each soldier carries with him. It is capable of cooking his rations either as a fry or a stew, the lid serving as a frying-pan and the lower part, which holds a quart of water, for stewing purposes. In the use of his mess-tin as a cooking utensil every soldier is trained, and the plan followed is to arrange the tins in a "kitchen" of eight with a fire in between. The form of cooking chosen is that of a stew, the meat being cut up, mixed with vegetables, covered with water and seasoned. From one to one and a half hours will be needed for cooking such a dinner, and it will be necessary to change the position of the tins in each "kitchen" from time to time, as some will be cooked sooner than others. With attention to details the mess-tin serves as an admirable cooking utensil, and it has the further recommendation that it economises fuel.

### Special Arrangements.

We have gone somewhat fully into the details of field cooking as carried out in the Regular Army because it is the type that the Territorial Force requires to be conversant with, seeing that its annual training is always done in camp, and if called up in case of invasion it would be constantly in the field. Further, the arrangements in vogue in the Territorial Force are on the same lines as those already described. Every unit has its certificated sergeant-cook, with one or two men per company, according to strength, as assistant cooks, and service kettles are issued to it for camp on the same scale as to a regular unit. In addition, it has a Soyer's stove, that is available for stewing, or boiling water, or making tea. Many of the regiments have provided themselves with special stoves to supplement the regulation equipment. These undoubtedly add to the comfort of the men in camp by giving a greater variety in their food, but at every camp care should be taken that the cooks are made proficient in the use of the recognised service kettles issued to them, and that the men have instruction given to them in the proper way of utilising their mess-tins as cooking utensils should the conditions of service call for this being done. In the Field Ambulances the cooking requirements are somewhat different, as a field ambulance has to cook both for the men belonging to it and also for the patients in it. For these latter special cooking is needed, and we find the regulations lay it down that every tent division must have three corporals and three privates with a knowledge of sick cookery. This gives a corporal and a private to each section. In addition, every field ambulance is furnished with special apparatus for invalid cookery, and of this we purpose giving an account in another article. Meanwhile we would only emphasise the necessity for scrupulous cleanliness in everything connected with cooking arrangements in camp and the need for furnishing the cooks and attendants with ample facilities for hand ablution in close proximity to their work.

